# Innovative Approaches and Evolving Strategies in Heavy Metal Bioremediation: Current Limitations and Future Opportunities

**DOI:** 10.3390/jox15030063

**Published:** 2025-04-26

**Authors:** Cristina Firincă, Lucian-Gabriel Zamfir, Mariana Constantin, Iuliana Răut, Maria-Luiza Jecu, Mihaela Doni, Ana-Maria Gurban, Tatiana Eugenia Șesan

**Affiliations:** 1 Biotechnology Department, National Institute for Research & Development in Chemistry and Petrochemistry—ICECHIM, 202 Spl. Independenței, 060021 Bucharest, Romania; cristina.firinca@icechim.ro (C.F.);; 2 Department of Botany and Microbiology, Faculty of Biology, University of Bucharest, 91–95 Spl. Independenței, 050095 Bucharest, Romania; 3 Field Crop Section, Academy of Agricultural and Forestry Sciences, Bd Maraști 61, 011464 Bucharest, Romania

**Keywords:** bioremediation, heavy metals, genetic engineering, nanotechnology

## Abstract

Decades of technological advancements have led to major environmental concerns, particularly the bioaccumulation of heavy metals, which pose persistent risks to ecosystems and human health. Consequently, research has increasingly shifted from conventional remediation techniques toward more sustainable, environmentally friendly solutions. This review explores recent advancements, ongoing challenges, and future perspectives in the field of bioremediation, emphasizing its potential as a green technology for heavy metal decontamination. Despite significant progress, key challenges remain, including scalability limitations and the management of bioremediation by-products, along with the influence of regulatory policies and public perception on its large-scale implementation. Emerging approaches such as genetic engineering and nanotechnology show promise in overcoming these limitations. Gene editing allows the tailoring of specific metabolic traits for bioprocesses targeted towards increased tolerance to pollutants and higher biodegradation efficiency, higher enzymatic specificity and affinity, and improved yield and fitness in plants. Nanotechnologies, particularly biogenic nanostructures, open up the possibility of repurposing waste materials as well as harnessing the advantages of the biosynthesis of NPs with higher stability, biocompatibility, and biostimulant capacities. Furthermore, biopolymers and bio-based nanocomposites can improve the efficiency and costs of bioremediation protocols. Even so, further research is essential to evaluate their long-term risks and feasibility.

## 1. Introduction

The global concern over heavy metal pollution stems from their non-biodegradable nature, thus leading to their prolonged existence in various environmental compartments such as soil, water, and biota. The accumulation of biologically available fractions in the tissues and organs of living organisms exerts numerous toxicological effects, ultimately representing a major concern for human safety [1,2]. Heavy metals are constituents of the lithosphere which can be released naturally through pedogenic and volcanic processes (e.g., geological weathering, soil erosion, atmospheric deposition, and volcanic eruption), but more significantly through anthropogenic activities (e.g., mining, smelting, industrial processing, agrochemicals, constructions, and improper maintenance of landfills) and accidental events [3]. Numerous historical incidents have proven the risks associated with heavy metal contamination, such as the Hinkley groundwater contamination with hexavalent chromium [4], the arsenic water contamination in Bangladesh [5], and the mercury poisonings in Minamata, Japan and Grassy Narrows, California [6], as well as the Bunker Hill lead poisoning [7].

The general consensus defines heavy metals based on biological, chemical, physical and toxicological properties as metals and metalloids with a density above 5 g∙cm^−3^, with potential toxic effect on living organisms [8]. However, the controversy still persists regarding a more comprehensive definition, their significant toxicity at low doses of exposure being considered the most important criterion compared to the chemical profile. In this sense, arsenic is often included in the category of heavy metals, although it is defined as a metalloid, exhibiting both metallic and non-metallic properties. In this paper, for consistency, we have maintained the general term of “heavy metal”, including this element [8]. Based on their physiological importance, they are differentiated as essential and non-essential. Essential elements are indispensable for the optimal functioning of organisms, because an insufficient intake consistently results in a reduction in a biological function from optimal to suboptimal. Supplementation with physiological levels of this element—and not others—can prevent or remedy this dysfunction. Essential metals such as zinc (Zn), iron (Fe), magnesium (Mg), copper (Cu), manganese (Mn), and trivalent chromium (Cr^3+^) act as cofactors of metalloenzymes, deficiencies or excesses of these metals leading to systemic dysfunctionalities [9]. These metals, also regarded as micronutrients, are involved in various important processes such as photosynthesis, respiration, and chlorophyll production in plants [10], DNA replication and transcription, the synthesis of neurotransmitters, electron transfer, energy metabolism, oxygen storage, and antioxidant defense in mammals [11], and microbial fitness, synthesis, and the functionality of biomolecules such as proteins, as well as cellular signaling and intracellular transport, in microorganisms [12]. On the other hand, non-essential metals such as hexavalent chromium (Cr^6+^), mercury (Hg), arsenic (As), cadmium (Cd), and lead (Pb) lack biological functions and pose significant toxic effects even at low doses of exposure [13].

Exposure to non-essential heavy metals has been directly associated with carcinogenicity, organ damage, severe systemic abnormalities, and even mortality [14]. Disruptions are observed from lower trophic levels, such as primary producers (vascular and non-vascular plants and algae) and decomposers (microorganisms and invertebrate organisms), to higher trophic levels, the effects being amplified along the food web [15]. The diversity, structure, and metabolism of the microbial community are affected, which in turn inhibits organic matter mineralization, nutrient cycling, and plant growth [16]. In plants, elevated levels of heavy metals impact morphology, growth, development, and important metabolic functions such as photosynthesis, respiration, and nutrient uptake [17]. As a consequence, higher trophic levels are affected through bioaccumulation and biomagnification, with effects being observed in the form of malformations, decreased survival and reproduction rates, and impaired development rates and physiological functionality [18]. The negative impact of heavy metals not only disrupts biological diversity and environmental processes but also alters ecosystemic structures. Soil and water quality degradation is observed through modifications in physical and chemical parameters that further affect the local biological communities [19].

Although heavy metal emissions have decreased in developed countries in the past century [20], active heavy metal contamination is still present in the environment decades later, affecting all forms of life. Extensive research has been conducted in recent years on biogeochemical cycles, health risk assessment, and remediation options for heavy metal contamination. Thus, it is essential to have an overview of research trends and future directions on this topic of major importance in order to better understand the opportunities for advancing the limitation and remediation of contamination with these xenobiotic compounds.

This review aims to explore the challenges posed by heavy metal contamination in different environments and assess the current advancements in bioremediation strategies. The approach to the subject takes into account the following objectives: (i) Presenting current methods used in the bioremediation of non-essential heavy metals, namely arsenic, lead, cobalt, cadmium, hexavalent chromium, mercury, and nickel, including the biological mechanisms involved and limitations in applicability; (ii) exploring innovative strategies, such as the use of genetically modified microorganisms and plants, as well as nanotechnology-based techniques; and (iii) identifying future opportunities for optimizing and expanding the applicability of bioremediation, including integration with other remediation technologies and the development of sustainable solutions.

Additionally, this review discusses technological and regulatory barriers, as well as future perspectives on improving bioremediation’s efficiency through genetic engineering, nanotechnology, and integrated remediation techniques, in order to enhance its applicability in real-world scenarios. In order to have a clear image of the progress made in the field of heavy metal bioremediation and to identify future development directions, a detailed analysis of the existing scientific literature is essential. In this regard, the following subsection briefly presents the most relevant publications on this topic, highlighting the current trends, reported challenges, and the solutions proposed by researchers.

### Research Publications on Heavy Metals

For the scope of this article, a systematic literature survey was conducted on the PubMed, Google Scholar, and Scopus databases for research articles, reviews, and books published in the past decade. The review process involved the selection of relevant articles based on well-suited keywords.

Publications were selected and analyzed on the basis of the relevance to the topic, the methodologies employed, the results and observations formulated, and the year of publication. The main criteria taken into consideration were articles containing comparative studies on already established biological remediation methods, studies on novel and innovative traits to increase the efficiency of technologies for up-scaling and field applications, and articles highlighting gaps and limitations identified in their practice.

**Figure 1:**
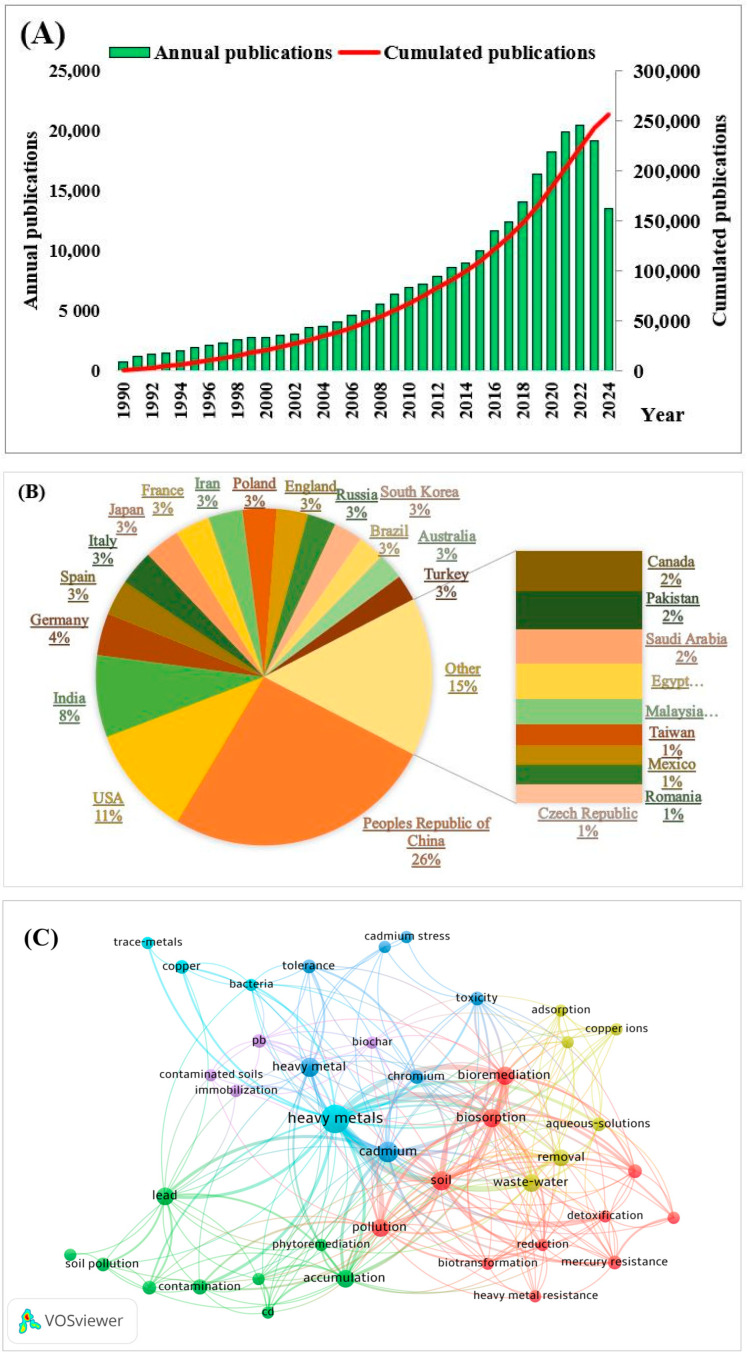
(**A**) Number of publications per year along with cumulated annual publications in the Web of Science database citing the term “heavy metal” between 1990 and 2024. (**B**) First 25 countries, based on total number of publications in the Web of Science database, citing the term “heavy metals” between 1990 and 2024. (**C**) Network visualization on co-occurrence of keywords related to heavy metals, generated using the VOSviewer software (version 1.6.20, The Netherlands).

A bibliometric analysis of scientific publications issued between 1990–2024 was conducted on the Web of Science (WOS) database regarding research carried on heavy metal contamination over the last decade (Figure 1). VOSviewer (version 1.6.20, The Netherlands) was used to construct a network visualization map based on data obtained from the selected database.

As indicated in Figure 1A, in the span of 34 years, a total of 256,152 papers were published on the subject of “heavy metals”. Research has steadily increased, exceeding 10,000 publications per year starting in 2015. In 2022, this productivity had doubled, exceeding 20,000 publications per year. The countries with the highest number of published papers are China (20.5%), the USA (8%), and India (6%), which are not only major economies involved in metal mining and smelting [21] but also face significant economic, environmental, and health casualties as a cause of heavy metal pollution [22]. The network visualization in Figure 1C divides data by clusters, connected through links. The nodes are represented by keywords, and these are connected to each cluster, differentiated by color. The line between ndes represents the strength of association between keywords, thicker lines indicating a higher frequency of co-occurrence. The criterion of co-occurrence for a keyword was determined to be its appearance at least twice in different publications.

We identified 386 keywords, out of which 43 met the established threshold of co-occurrence. The highest co-occurrence and consequently the strongest links were observed for the general concept of heavy metals, followed by soil, biosorption, bioremediation, pollution, and specific metals such as cadmium, chromium, and lead, indicating a growing interest for biological remediation methods as well as a continuous demand to expand the knowledge on heavy metal pollution. There is also an increasing interest in studying the potential of specific microbial strains and plants for the remediation of contaminated sites, as well as their particular tolerance mechanisms and metabolic pathways.

In Romania, a total of 2807 papers have been published in the last 34 years, with a steady increase starting in 2009 and becoming even more significant since 2021. Studies were focused primarily in fields such as environmental science, chemistry, and engineering, highlighting the interest for developing new materials and technologies in a sustainable manner. In that sense, there is an available niche nationally for better understanding and refining biological remediation methods in order to align with the global intention of environmental restoration and sustainable development goals.

This study presents a comprehensive review of the current biological remediation methods for heavy metal-contaminated terrestrial and aquatic environments, with a focus on upscaling and field applications for long-term restoration. To achieve this, a systematic literature survey was conducted using PubMed, Google Scholar, and Scopus, covering research articles, reviews, and books published over the past decade. The selection criteria prioritized comparative studies on established bioremediation methods, innovative approaches for enhancing efficiency in large-scale applications, and analyses highlighting existing gaps and limitations. The bibliometric analysis underscores the increasing scientific interest in heavy metal contamination and the development of sustainable, eco-friendly remediation strategies. As such, this study contributes to the field by advancing current knowledge and providing a foundation for broader applications, including the implementation of novel technologies such as genetic engineering and nano-based approaches. This bibliometric analysis highlights the growing scientific attention to the issue of heavy metal contamination of the environment, as well as to the most sustainable and environmentally friendly methods for its remediation. Bioremediation represents an ecological and sustainable approach to the mitigation of heavy metal contamination. Our study contributes to this growing field by evaluating the efficiency of microbial-based remediation strategies, demonstrating their potential in reducing environmental contamination and restoring ecosystem balance.

The analyzed publications highlight the critical role of bioremediation in addressing heavy metal contamination, providing a viable alternative to conventional methods, which are often costly and often environmentally aggressive. This research not only advances current knowledge but also provides a basis for optimizing bioremediation processes through improved microbial selection, genetic modification, and integrated remediation strategies.

## 2. Heavy Metals Occurrence and Toxicity in the Environment

Heavy metals are classified as xenobiotics in toxicology and environmental science, requiring specialized removal strategies at the individual, population, and ecosystemic levels due to their accumulation and persistence, which leads to chronic toxicity. Unlike organic xenobiotics (e.g., synthetic pesticides or pharmaceuticals), heavy metals do not degrade under the action of abiotic or biotic factors, tending to bioaccumulate and be further amplified and transformed along food chains [23].

**Figure 2:**
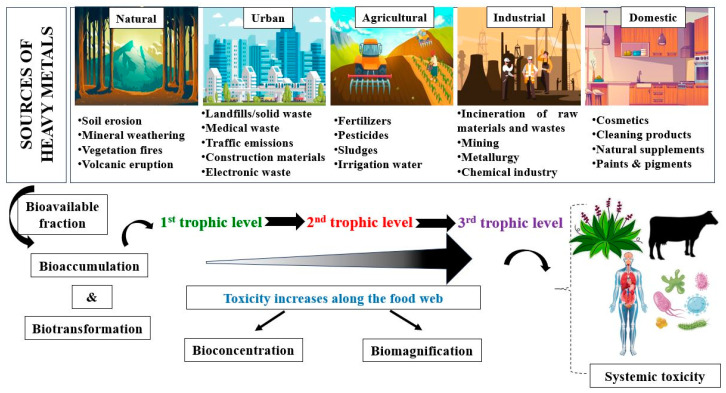
Schematic representation of the biogeochemical cycle of heavy metals in the environment. (figure created using Vecteezy graphics).

Anthropogenic sources of domestic, industrial, commercial, and agrochemical wastes represent the primary pathway of heavy metal release in the natural reservoirs [24]. Within the trophic levels, these hazardous compounds may be bioaccumulated, bioconcentrated, and biomagnified, altering ecosystemic quality and threatening the safety of all living organisms, including the human population (Figure 2) [25].

In aquatic environments, heavy metals can be accumulated in sediments and suspended solids or easily absorbed by aquatic fauna and flora [26]. In soil, they can be transported through leaching into groundwater and surface water and can be assimilated by the local biodiversity. The bioavailability of metals to living organisms is dictated by the physical, chemical, and geochemical particularities of the metal, as well as the biological affinity of the organism [27]. Human exposure generally occurs through the ingestion of contaminated water or food, inhalation, or dermal contact, either in an occupational, household, or accidental context [28].

Heavy metals can disrupt normal cellular functions when introduced into living organisms by binding to proteins, enzymes, and DNA, thereby disrupting metabolism, oxidative stress balance, and cellular signaling. They often mimic essential elements, leading to improper biochemical interactions (e.g., lead replacing calcium in bones), exposure being linked to neurological damage (e.g., mercury and lead), carcinogenicity (e.g., arsenic and cadmium), and organ failure [29]. Essential heavy metals may also exert toxic effects if their concentrations exceed the thresholds of tolerance. For instance, zinc is indispensable for several enzymatic activities and cellular functions, having a protective effect against inflammation, oxidative stress, and genotoxicity at physiological levels. However, chronic elevated levels cause impaired immune function, neuronal diseases, and even carcinogenicity [30].

Nevertheless, non-essential metals pose higher risks due to their severe toxicity even at low concentrations of exposure, with detrimental effects on normal physiological functions. Thus, arsenic, hexavalent chromium, nickel, and cadmium are classified as group 1 carcinogens by The International Agency for Research on Cancer (IARC), whereas mercury, lead, and cobalt are recognized as potentially carcinogenic [31]. Their concentration is strictly regulated in drinking water and agricultural soil (Table 1) [32,33,34].

Furthermore, several International Conventions and Programs have been implemented through the United Nations (UNEP) and other international agencies for the purpose of monitoring, limiting, and preventing pollution with heavy metals and their compounds [35].

Below, some general toxicological aspects of the principal non-essential heavy metals and metalloids will be briefly explained.

Arsenic is ranked first on the Priority List of Hazardous Substances according to the United States Agency for Toxic Substances and Disease Registry (ATSDR) [36]. Inorganic compounds, such as trivalent arsenite (As^3+^) and pentavalent arsenate (As^5+^), are significantly more toxic compared to organic forms [37]. Their chronic presence in blood of concentrations exceeding 0.1 µg/L can lead to a condition described as arsenicosis, associated with dermatological, cardiovascular, hematological, neurologic, respiratory, and gastrointestinal dysfunctions which can progress towards complications of malignant nature [38].

Lead is recognized as the second most toxic metal after arsenic due to its genotoxicity and carcinogenic potential [39]. Its long biological half-life of up to 15 years in the bone matrix and extensive utility in commercial products [40] upholds the importance that it has been granted over the years. An enhanced concentration in blood, exceeding 0.5 µg/L, induces a broad range of physiological, biochemical, and morphological effects, characterized by behavioral, cognitive, and neurological impairment, prenatal and postnatal defects, and cardiovascular, renal, reproductive, neuro-muscular, and skeletal impaired functionality [41].

Although nickel is regarded as an essential micronutrient involved in several biochemical and physiological processes for plants, animals, and microorganisms [42], systemic toxicity in humans has been observed for blood concentrations exceeding 50 µg/L [43]. Acute exposure manifests though gastrointestinal symptoms, followed by delayed effects such as respiratory, endocrine, cardiovascular, and neurological disorders, with long-term accumulation leading to immunotoxicity, genotoxicity, and carcinogenicity [44].

Mercury, especially methylmercury (MeHg) and elemental mercury (Hg^0^), is associated with neuropathological symptoms which tend to be present more significantly in vulnerable categories, especially among children [45]. For this reason, the World Health Organization (WHO) and the U.S Food and Drug Administration (FDA) specify clear recommendations of clinical intervention in the case of a hair-to-blood concentration ratio of 250:1 and blood concentration above 40 µg/L [46].

Cadmium, with a long biological half-life estimated to be up to 30 years [47], can exert significant toxicity observable at blood concentrations above 0.4 µg/L. Damage of several organs can be induced by acute exposure, as well as reproduction dysfunctions and fetal development disorders, which can progress to pulmonary, renal, and bone lesions, ultimately exerting severe teratogenic and carcinogenic effects [48].

Chromium, in its trivalent form (Cr^3+^), has been considered an essential nutrient involved in glucose and lipid metabolism [49]. However, long-term or excessive supplementation may inhibit iron and zinc absorption, alter calcium metabolism at the bone level, and induce genotoxic effects [50]. Hexavalent chromium, on the other hand, is recognized as highly toxic, with mutagenic, teratogenic, and carcinogenic potential at blood concentrations exceeding 0.5 µg/L. It can easily pass through cell membranes due to the resemblance to SO_4_^2−^ and PO_4_^2−^ ions, where it is reduced to Cr^5+^, Cr^4+^, and ultimately Cr^3+^, simultaneously generating free radicals and inducing oxidative stress [51].

Cobalt is an essential constituent of vitamin B12 and a coenzyme involved in numerous biological processes [52]. Acute toxicity often results from excess supplementation or from exposure to its inorganic forms, with effects being observed at concentrations above 300 µg/L in blood [53]. Chronic exposure leads to a condition known as cobaltism, inducing toxicity in the cardiovascular, hematological, respiratory, endocrine, and neurological systems, further progressing towards cytotoxicity, genotoxicity, and carcinogenicity [54].

**Figure 3:**
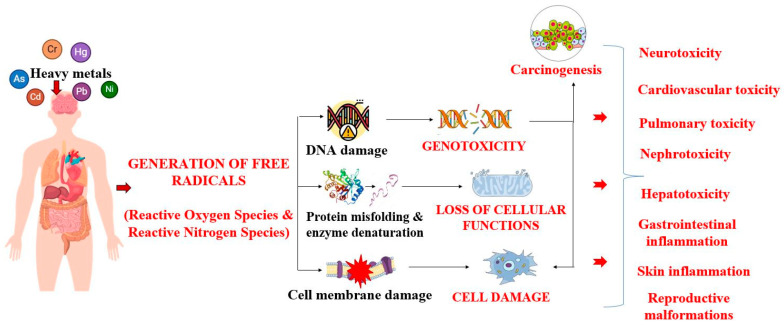
Mechanisms of action and toxicity of heavy metal accumulation in the human body. Figure created in the Mind the Graph platform.

Heavy metal toxicity manifests extensively on the cellular, tissue, and organ levels. The primary mechanism of toxicity is through the oxidative deterioration of biological macromolecules due to binding to nuclear constituents (Figure 3) [55,56].

During this process, reactive oxygen species (ROS) and reactive nitrogen species (RNS) are produced, while intracellular antioxidants and enzymes are inhibited, impairing normal metabolic functions [55]. Furthermore, the significant dysregulation of cellular growth and differentiation and DNA repair, disruption of protein signaling, and activation of transcription factors eventually induce carcinogenic effects [56].

In mammals, metals are generally deposited in muscles, bones, and adipose tissues and oftentimes impair the uptake and metabolism of other minerals, disrupting the body’s homeostasis [57]. Due to their biophysiological similarities with essential nutrients such as zinc, calcium, magnesium, and iron, toxic metals can compete with metal ions for binding sites and displace them in their specific biological structures. Concurrently, deficiencies in essential metals can enhance the absorption of toxic elements [58]. Studies conducted by Brodziak-Dopierała [59] and Chen et al. [60] revealed a direct association between lead and cadmium accumulation, respectively, and a decrease in calcium deposition in bones, with higher incidences of chronic pain, osteoporosis, and fractures. Lead and cadmium also interfere with iron metabolism by downregulating the expression of genes responsible for iron transport, which decreases its accumulation [61].

The speciation, bioavailability, and concentration of exposure influence the toxicological effects of heavy metals, and as such, their accumulation in the environment poses numerous risks to all forms of life, creating the requirement for efficient remediation and restoration protocols. Thereafter, we will present the principal bioremediation technologies and their advantages over conventional treatment methods.

## 3. Biological Remediation Techniques Overview

The evolution of emerging pollutants and the resulting environmental deterioration represent a matter of critical importance which requires the development and implementation of sustainable, cost-effective, and efficient technologies. To date, up to 350,000 new compounds and chemical compositions have been created, being detected in even the most remote of places [62]. Currently, several options applicable in situ and ex situ are available for the remediation of aquatic and terrestrial systems. These methods can be categorized as physical, chemical, and biological (Table 2). Oftentimes, a singular technology is not completely efficient in removing the targeted contaminants, requiring a combination of synergistic methods in pair with monitoring and improving strategies to coordinate with the complexity of natural systems and the level of pollution [63].

Physical and chemical remediation technologies have the advantages of simple, controllable applications that can prevent heavy metal migration, most of them being applied presently and with a long-term understanding of the mechanisms. However, the main limitations are the production of secondary wastes and alteration of environmental structure and functions, while certain methods may not be fully effective in removing complex, significant levels of pollution [64,65,66,67,68,69].

Furthermore, their efficiency is dictated by environmental conditions, requiring frequent monitoring and adjustments, which can involve higher costs and use of resources [70]. Biological methods, on the other hand, involve the use of the natural mechanisms of accumulation, uptake, and metabolism possessed by biological systems, namely bacteria, fungi, microalgae, and plants, for the removal of pollutants from various natural or artificial settings [71]. Such an approach is more beneficial compared to conventional methods due to its low costs, higher acceptance by decisionmakers and the general public, facile in situ approach with minimal environmental impact, and potential for ecological conservation and restoration [72]. Biological remediation methods can be employed either in situ or ex situ and are categorized as either microbial bioremediation or phytoremediation based on the primary organisms used.

### 3.1. Bioremediation

**Figure 4:**
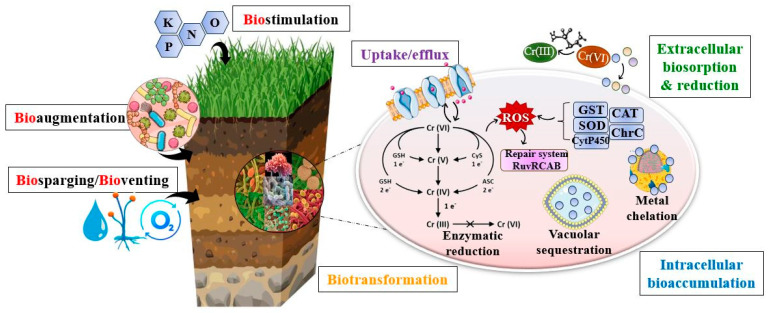
Bioremediation mechanisms employed by microorganisms in the presence of heavy metals.

Bioremediation describes the use of microorganisms for the stabilization and removal of pollutants, and as such may be intrinsic or engineered. Intrinsic bioremediation, or natural reduction, involves the passive remediation of polluted sites by the endemic microbiota without human involvement [73] and is particularly suited for low levels of contamination. Engineered bioremediation relies on human intervention to optimize ecological factors, improving microbial growth and metabolic activity, thus being able to address more severe contamination [74]. Among the mechanisms employed by microorganisms, the main ones are bioaccumulation, biosorption, and biotransformation (Figure 4).

Biosorption encompasses the uptake of metal ions from the environment through entrapment, ion exchange, chelation, complexation, and micro-precipitation. Biological factors such as cell wall structure and cell physiology, as well as physicochemical factors, determine biosorption capacity. Generally, biosorption is enhanced in alkaline conditions and relatively high temperatures (up to 35 °C), conditions that improve the affinity of metal ions for the binding sites available onto the microbial cell [75]. Biosorption can occur using either living or dead (inactivated) biomass, the latter being considered superior in terms of the possibility of metal recovery, reusability, and maintenance requirements for the used biomass. However, adsorption capacity is significantly higher in live biomass due to active uptake mechanisms in addition to passive adsorption [76]. Once within the cell, metal ions become subjected to bioaccumulation and biotransformation.

Bioaccumulation occurs when the rate of absorption of contaminants is above the rate of its elimination from the system, with compounds being bound to intracellular structures. Initially, metal ions are attached to the cellular surface, with minimal metabolic involvement. Afterwards, they are distributed throughout the cell membrane, ultimately being either stored in cytoplasmatic structures such as vacuoles or in coordination with cytosolic metal-binding proteins [77]. The most widely studied proteins of such importance are metallothioneins, which are cysteine-rich proteins that are induced in the presence of heavy metals. The ability to synthesize these proteins has been proven in numerous microbial strains such as *Escherichia coli*, *Saccharomyces cerevisiae*, and *Candida* sp., as well as *Penicillium* sp. [78,79,80,81].

Biotransformation is a resistance mechanism involving the chemical alteration of hazardous compounds through oxido-reduction, methylation, and demethylation, leading to the synthesis of less toxic forms by the enzymatic activity of reductases and methyltransferases. For instance, the enzymatic reduction of Hg^2+^ to HgO has been obtained by using bacterial mercury reductases, whereas methyl-, phenyl-, and ethyl-mercury have been proven to be metabolized by organomercurial lyases, forming Hg^2+^ along with either methane, benzene, or ethane [82]. In regards to chromium biotransformation, chromate reductases are the main agents involved in the reduction of Cr(VI) to the less toxic Cr(III), their activity being observed in both bacteria and fungi [83]. However, biotransformation may sometimes lead to the formation of chemical forms with amplified toxicity compared with the parent material. The reduction of Cr(VI) results in the short-term formation of reactive intermediates such as Cr(V) and Cr(IV) that can oftentimes generate the production of ROS and even induce DNA breakage [84]. The process of biotransformation and microbial metabolism in general can be enhanced through the addition of glucose and other organic amendments which facilitate the reduction, stabilization, and decrease in bioavailability of metals [85].

Table 3 presents the bioremediation efficiency obtained by using different microorganisms applied to natural or synthetic media contaminated with heavy metals under laboratory conditions [86,87,88,89,90,91,92,93,94,95,96,97,98,99,100,101,102].

In addition to the natural metabolic adaptations of microorganisms to heavy metal contamination, the bioremediation process can be enhanced in situ through bioaugmentation/biostimulation, bioslurping, bioventing, and biosparging.

Bioaugmentation involves the addition of pre-grown endogenous or exogenous microbial strains, either in their natural state or genetically engineered, that have proven metabolic efficiency for the desired purposes [103]. Biostimulation, on the other hand, implies supplementing nutrients and electron acceptors to enhance intrinsic microbial metabolism [104].

Bioventing is another form of stimulating the intrinsic microbial population through supplying oxygen into the vadose zone, with limitations in soils high in moisture or low gas permeability [105]. For groundwater remediation, biological reactive barriers, bioslurping, and biosparging are often employed. Biological barriers, also known as permeable reactive bio-barriers are intended to capture and facilitate the degradation of pollutants while allowing groundwater flow [106]. On the other hand, biosparging and bioslurping are frequently used in combination and involve the injection of pressurized gaseous components into the saturated zone, leading to the volatilization of contaminants which are then transported to the unsaturated zone with the displaced pore water. The addition of oxygen also supports aerobic biodegradation, but the method is limited to a depth of 25 feet below the surface [105].

Bioremediation can be carried out ex situ as well, through bioreactors, biopiles, and land farming. Although more costly compared with the in situ approach, this can be effective in treating large quantities of contaminated soil or water.

Landfarming is a method that treats the surface layer by tilling or plowing, thus aerating the soil and stimulating the intrinsic biodegradation process. Depending on the depth of the polluted site, it can be applied in situ as well. Similarly, biopiles combine landfarming with composting, mixing various amendments into the excavated soil [107]. Bioreactors are recognized as the most favorable ex situ remediation technique, used for the treatment of recalcitrant compounds in both solid and slurry phases, the process being easily controlled and automated and therefore significantly enhancing microbial biodegradation. The primary limitation stems from the disposal and further need for treatment of the resulting effluent [108].

### 3.2. Phytoremediation

The concept of using plants for the purpose of removing xenobiotic compounds from the environment was introduced as “phytoremediation” in 1983 by R.L. Chaney [109] based on the principle that certain plants are able to accumulate high concentrations of metals in their roots and aerial organs. Prolonged exposure to increased levels of pollution can determine the adaption of individuals, exhibiting a variety of physiological responses. Therefore, plants may be categorized as bioindicators, excluders, or accumulators. Bioindicators are highly sensitive to environmental stressors and as such are used as pollution markers, providing information about the population’s level of exposure through specific morphological alterations and concentrations present in plant tissues [110]. Excluders, on the other hand, restrict the concentration of pollutants that can be uptaken within the radicular system and translocated into their biomass, thus protecting the aerial organs and physiological integrity of the plant. Accumulators pose the capacity to absorb pollutants into their above-ground organs at levels that do not significantly dysregulate growth and development, but that are still higher than those withstood by the other categories. In the category of accumulators, plants that are able to accumulate concentrations above a certain threshold are deemed hyperaccumulators [111]. The principal mechanisms that distinguish hyperaccumulators from regular plants are efficient uptake through the root symplast, enhanced translocation from the rhizosphere to above-ground structures and superior metabolic adaptations against oxidative stress. Due to their genetic adaptability, hyperaccumulators are deemed more favorable for phytoremediation purposes [112].

A Global Hyperaccumulator Database (GHD) has been created, containing over 700 plant species to date that have been confirmed as efficient in heavy metal phytoremediation, making up only approximately 0.2% of identified vascular species [113]. In this database, only plants that meet the following criteria are included: able to uptake >100 µg/g cadmium, selenium, or thallium, >300 µg/g chromium, cobalt, or copper, >1000 µg/g arsenic, lead, or nickel, >3000 µg/g zinc, or >10,000 µg/g manganese [114]. It is important to note that invasive species and plants of agronomic importance such as medicinal plants, aromatics and edible crops are also used in phytoremediation, which may pose long-term risks to human safety and environmental integrity.

**Figure 5:**
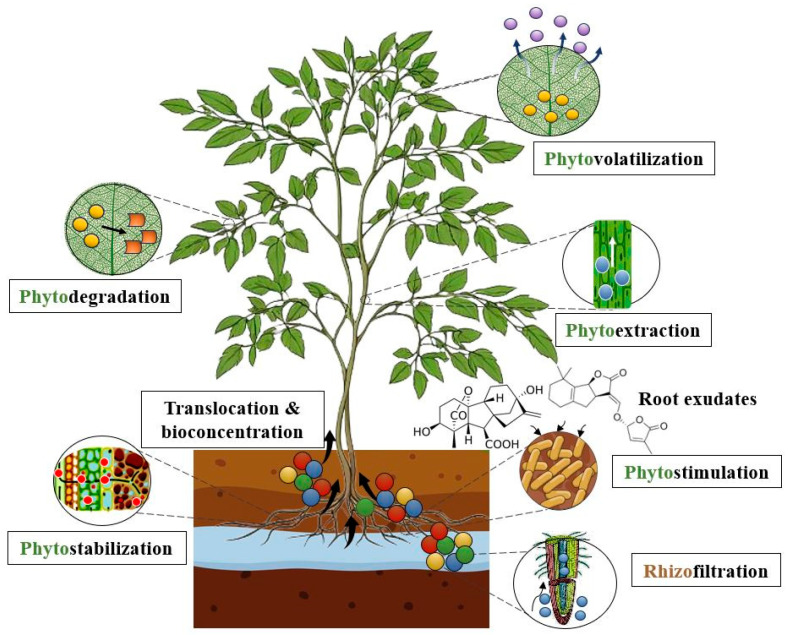
Phytoremediation mechanisms employed by plants in the presence of heavy metals (Figure created in the Mind the Graph platform).

Phytoremediation may be employed in both aquatic and terrestrial environments through rhizofiltration, phytoextraction, phytovolatilization, phytostabilization and phytotransformation. These processes can also be supported through by the symbiotic relation between plants and the soil microbiome through phytostimulation [115] (Figure 5). Certain functional traits seem to be more favorable in phytoremediation, as studies demonstrate that dense root systems with a higher number of young, fine roots, higher leaf biomass, higher growth rate, and greater overall biomass production are correlated with a higher bioremoval efficiency against heavy metals [116]. Environmental parameters such as pH, temperature, organic matter content, soil texture, and nutrient availability also directly influence phytoremediation by dictating the speciation, bioavailability, and mobility of metal ions, as well as microbial diversity and functions, crop yield, and plant metabolism [117].

Within the soil, heavy metals may be stabilized through phytostabilization by a variety of root exudates produced by plants which form stable complexes with metal ions at the rhizosphere level, thus restricting uptake into root cells. These compounds contain polysaccharides, peptides, proteins, enzymes, and organic acids that also support the metabolism of indigenous microorganisms, thus enhancing microbial biodegradation [118]. Plants can sequester heavy metals within their root system or bind them to the soil matrix, effectively reducing their migration into groundwater or uptake by adjacent biota. This is particularly beneficial in terrestrial environments, further preventing erosion, improving soil fertility, and enabling the long-term remediation of the contaminated environments [119].

Uptake is mediated through phytoextraction, metal ions being further translocated throughout plant organs, with compartmentation in metabolically inactive components of plant tissues such as the cellular wall, membrane, and vacuoles [120]. This mechanism is thought to be efficient in the permanent removal of heavy metals from soil and aqueous environments [121]. Once within the plant cells, metal ions go through a process of phytotransformation, or phytodegradation, involving the metabolization of pollutants through various enzymatic reactions and metabolic processes [122]. Similarly, phytovolatilization converts pollutants into volatile, less toxic forms that are then released into the atmosphere. For instance, trimethylarsine, which, although less toxic than the parent metal, may still be potentially hazardous [123,124]. It is considered to be a controversial method, as the volatilized compounds can be redeposited in the environment, and as such, the general consensus is that it should not be utilized broadly [125]. Phytofiltration removes pollutants through adsorption, concentration, and precipitation using roots (rhizofiltration), shoots (caulofiltration), or seedlings (blastofiltration) [126]. The process is similar in mechanism to phytoextraction; however, its main applicability is in groundwater and surface water systems. Aquatic macrophytes or hydroponically cultivated terrestrial plants may be acclimatized and used either in situ, or ex situ in tank systems or pump and treat systems that make contaminated groundwater available to above-ground systems [127].

Some examples of natural hyperaccumulators, high-biomass producing species, or genetically engineered species that can be used for the efficient accumulation of heavy metals from the environment are presented in Table 4 [128,129,130,131,132,133,134,135].

Species such as *Salix* spp. and *Populus* spp. that are known (hyper)accumulators have been successfully used for the extraction of heavy metals (e.g., cadmium, chromium, nickel, and lead) [136]. The phytoextraction process can be enhanced using plant growth-promoting bacteria or arbuscular mycorrhizal fungi [137]; however, it is important to take into consideration the significant influence of plant morphology and level of heavy metal contamination on the efficiency of the process. Regarding transgenic or genetically modified plants, they have emerged as a promising tool in agriculture and environmental remediation. These plants are engineered by transferring genes from other organisms or overexpressing already existing ones to provide new or improved phenotypic characters such as increased yield, nutritional value, pest resistance, tolerance to abiotic stressors, and improved bioremediation [138]. Despite their benefits, several concerns have been raised, related to ethical, ecological, and techno-economic issues, which will be discussed in depth in the following chapter.

Having established a comprehensive overview of biological remediation techniques, recent advances that are pushing the boundaries of bioremediation will now be presented. Building on the fundamental understanding of biological remediation, recent advances in bioremediation have introduced innovative approaches that considerably enhance the efficiency, specificity, and applicability to various challenges posed by environmental contamination with xenobiotics. Cutting-edge innovations are increasing efficiency, precision, and adaptability, paving the way for more effective and sustainable environmental restoration strategies.

## 4. Novel Approaches and Emerging Strategies

Biological remediation methods are feasible, sustainable, and environmentally friendly, compared with conventional technologies. While laboratory results are often promising, scaling up to field applications requires consideration for the complex ecological processes and biogeochemical interactions that may hinder the efficiency of the process. Recent advancements in bioremediation technologies have greatly improved the possibility of addressing heavy metal pollution in contaminated terrestrial and aquatic environments. Plants and microorganisms that have adapted to contaminated environments possess certain biochemical and physiological attributes that enable them to use xenobiotics as carbon sources and even reduce their toxicity, such as increased enzymatic stability and functionality in extreme environmental conditions and the production of specialized biomolecules such as metallothioneins and extracellular polymeric substances (EPS) [139].

Recent advancements in bioremediation technologies focus on augmenting the metabolic capabilities of target microbial strains and enhancing their efficacy through integrated methodologies and novel functional nanomaterials. These innovations contribute to more efficient recovery and ecological restoration of contaminated sites, simultaneously improving waste management strategies, reinforcing ecosystem functions, and increasing the credibility and acceptance of bioremediation among policymakers and the scientific community.

### 4.1. Genetic Engineering in Bioremediation

Genetic engineering techniques are considered a complementary tool that allows improving the tolerance, bioaccumulation, and biotransformation potential of microorganisms and plants for environments contaminated with heavy metals. Various -omics such as transcriptomics, proteomics, metagenomics, and high-throughput sequencing have been employed in order to discover or gain a better understanding on existing functional genes, degradation pathways, and resistance systems, as well as modify and design novel traits for specific purposes [140]. Genetically engineered microbes (GEMs) are microbial strains whose genetic code has been synthetically altered by introducing or cloning genes with the purpose of gaining desired metabolic activities with high specificity. Additionally, multiple functional genes or mutations may be introduced into the genome, commonly known as multi-functional GEMs (MG-GEMs), in order to secure survival and bioremoval efficiency in environments containing mixed pollutants [141].

For bioremediation purposes, multiple approaches are available, such as the modification of enzyme affinity and specificity, construction and regulation of specific pathways, and development of target bioprocesses [142]. Compared with wild microbial strains that can display a slower bioremoval rate of pollutants or can face difficulties when introduced in the native microbial communities of the contaminated environments, GEMs have the potential to be used in bioaugmentation, being engineered to produce specific enzymes both for the degradation of xenobiotics as well as supporting phytoremediation employed by associated plants [143]. However, safety studies have to be thoroughly assessed, as microorganisms naturally exchange genes by means of transduction, transformation, and conjugation and genes encoding heavy metal resistance can also be responsible for antibiotic resistance [144]. Genes encoding multi-metal resistance in *Escherichia coli* and *Salmonella* spp. have been observed to confer resistance to β-lactam, tetracycline, and sulfonamide as well [145].

Genome sequencing provides information of the genetic material expressed in living organisms both as a result of long-term contamination as well as throughout metabolic processes involved in bioaccumulation and biotransformation. Studies on a multi metal-resistant *Micrococcus luteus* strain isolated from industrial wastewater revealed numerous arsenic resistance genes, including *arsC1*, *arsC2*, and *ACR3*, as well as the *aioB* gene encoding arsenic oxidation. Several other resistance genes were identified for zinc, cadmium, nickel, and mercury, responsible for intracellular transport, efflux, and biotransformation to less toxic forms [146]. New strains are continuously identified as candidates for bioremediation in contaminated environments, either for individual or multiple categories of pollutants. *Cladophialophora exuberans* presented genes responsible for heavy metal homeostasis for copper and lead, along with a significant number of genes responsible for Carbohydrate-Active Enzymes (CAZYs), indicating potential for hydrocarbon bioremediation as well [147]. A novel *Geotrichum* sp. CS-67 strain with active removal efficiency for Cu(II), Zn(II), and Ni(II) was identified by He et al. [148], while comparative genomic analysis employed by Chi et al. [149] revealed the chromium bioremediation potential of a *Penicillium janthinellum* P1 strain possessing the *ChrA* and *ChrR* genes encoding chromium resistance and reduction from Cr(VI) to the less toxic Cr(III).

**Figure 6:**
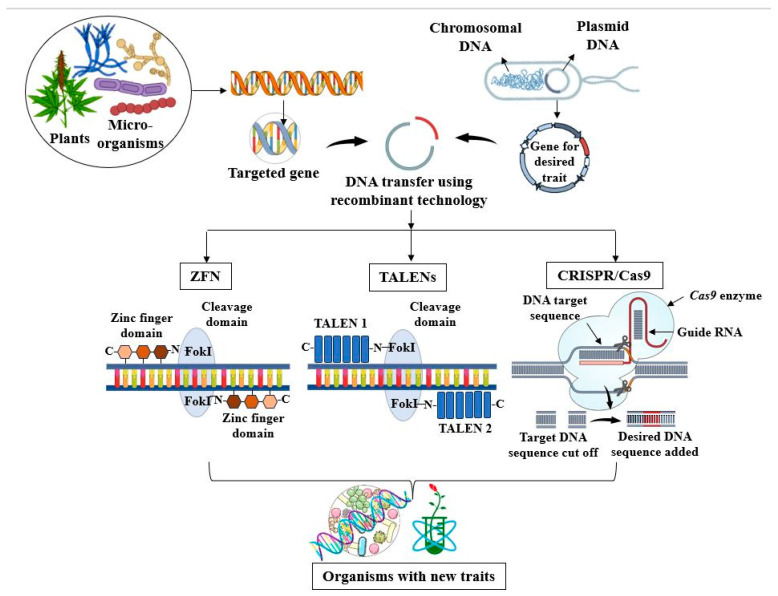
Gene editing technologies (zinc finger nucleases (ZFNs), transcription activator-like effector nucleases (TALENs), and (CRISPR)/CRISPR-associated endonuclease system (Cas)) used for improving targeted traits in plants or microorganisms (figure created in the Mind the Graph platform).

Emerging gene editing technologies, such as the CRISPR/CRISPR-associated short palindromic repeats (CRISPRs) system, transcription activator-like effector nucleases (TALENs), or zinc finger nucleases (ZFNs) (Figure 6) allow genome manipulation for multiple applications in medicine, agriculture, and biotechnology [150].

Through this approach, new functional traits can be obtained for the desired purposes and heavy metal resistance can be acquired in low-tolerant strains through gene transfer. *Escherichia coli* was engineered to express lead-binding proteins (*PbrR*) on its surface, significantly increasing lead uptake. Furthermore, the bacterial-immobilized lead presented lower bioavailability compared with oral exposure to Pb(II) [151]. Through genetic engineering, the S-adenosylmethionine methyltransferase gene (*CmarsM*) was transferred into *Bacillus subtilis* from the red alga *Cyanidioschyzon merolae* in order to methylate As(III) from contaminated manure, concomitantly ensuring arsenic resistance [152]. For efficient Cd(II) bioremoval, the *CadR* gene was transferred from *E. coli* to *Pseudomonas aeruginosa*, leading to the expression of metal binding proteins with high selectivity for cadmium and elevated adsorption capacity [153]. Additionally, mercury tolerance strains were transferred from resistant *Sinorhizobium medicae* to the nitrogen-fixing bacteria *Rhizobium leguminosarum*, conferring de novo adaptations to conditions of mercury pollution while simultaneously supporting plant growth and the nutrient pool in soil [154].

For phytoremediation, genetic engineering improves the uptake, translocation, and phytostabilization of xenobiotics, as well as the production of biomass and development of a more extensive root system. Transgenic plants also pose the benefit of resistance to pathogens, climate fluctuations, and lack of nutrients, thus improving yield and lowering the costs required for phytoremediation [155]. The most common method of genetic manipulation of plant genomes is through gene transfer mediated by the bacterial strains *Agrobacterium* spp. Strains belonging to this genus, especially *A. tumefaciens*, naturally transfer DNA sequences (T-DNA) into the plant genome, thus being considered a “natural genetic engineer”. First, plant-derived exudates activate the bacterial virulence system and consequent induction of virulence genes expression (*vir*), along with the generation of T-DNA. Then, the T-DNA attaches to vir-encoded proteins, which are exported out of the bacterial cell and transported into the host cell nucleus, where they become integrated into the plant chromosomal DNA [156]. This way, plants can acquire higher resistance to oxidative stress, alongside metal tolerance, accumulation, and detoxification capabilities [157].

Through the transfer of genes encoding metallothioneins, such as ChMTII, via *Agrobacterium*-mediated transformation, the ability of *Nicotiana tabacum* L. to reduce the concentration of lead was significantly increased, along with enhanced antioxidant production [158]. The tobacco plant has been intensely studied for its adaptation in diverse climatic conditions, facile genetic modification, and abundance of metabolites, thus becoming a model plant in research. Additionally, it is considered a good phytoremediation candidate for its quick development and biomass production, extensive root system, and efficiency in heavy metal uptake and translocation [159]. The plant has proven bioremoval efficiency in soil co-contaminated with cobalt, chromium, nickel, cadmium, and copper, in association with native rhizobacteria [160]. Besides the tobacco plant, there is an abundance of crops that have been subjected to gene editing technologies, primarily for improved resistance to climatic change and pathogens, such as *Arabidopsis thaliana*, *Nicotiniana tobacum*, *Triticum aestivum*, *Zea mays*, *Solanum lycopersicum*, *Sorphum* sp., and *Glycine max* [161]. However, they have been used for the phytoremediation of hazardous compounds such as heavy metals [162], pesticides [163], and endocrine disruptors [164] as well. In that sense, the continuous use of crops in bioremediation may pose the risk of reintroducing pollutants into the food web, additionally presenting a potential risk to human health through ingestion. For that reason, ornamental plants and species that are not generally intended for consumption have gained the interest of researchers. Members of the *Brassicaceae* family are often preferred for their rapid growth and development, which allows for multiple cultivation within a season, wide adaptation capabilities to environmental conditions, and high tolerance to increased concentrations of heavy metals [165]. About 25% of the members of this family have been identified as hyperaccumulators, with the highest number of nickel-accumulating plants from the entire group [165]. Species of the genera *Brassica*, *Noccaceae*, *Alyssum*, *Aravidopsis*, and *Thlaspi* have been proven to efficiently accumulate a variety of heavy metals such as cadmium, lead, chromium, nickel, and arsenic [166]. Furthermore, common ornamental plants such as *Celosia argentea*, *Amaranthus hypochondriacus*, *Calendula officinalis*, *Tagetes patula*, *Mirabilis jalapa*, or *Iris* sp. have been taken into consideration for their heavy metal hyperaccumulator potential [167]. Their short growth cycle, high biomass production, and broad tolerance to climatic conditions offer a promising opportunity for bioremediation, with minimal risks for the local biodiversity and even bringing economic benefits by conserving and supporting cultural heritage and ecotourism [168].

Both the United States and the European Union dictate strict regulations concerning introduced genetic material, prohibiting the introduction of genes that encode toxins or antibiotic resistance and imposing the limitation of transmission. In the case of European Union countries, the use of genetically modified organisms (GMOs) is regulated through the Directive 2009/41/EU, through the European Food Safety Authority (EFSA). A list of microorganisms regarded as safe is provided within the Qualified Presumption of Safety (QPS) concept, similar between the main bodies of authority, some examples being *Bifidobacterium* sp., *Lactobacillus* sp., *Bacillus* sp., *Debaryomyces* sp., *Saccharomyces* sp., and *Escherichia coli*, as well as *Aspergillus* sp., *Penicillium* sp., and *Trichoderma* sp. [169].

For transgenic plants, both EU and US regulations focus on rigorous environmental risk assessments, traceability, and labeling requirements, ensuring that genetically modified crops intended for food, feed, or cultivation need high safety standards. For EU countries, the authorization process is governed by Regulation (EC) No 1829/2003 and Directive 2001/18/EC, with decision-making involving both EFSA and EU Member States. Furthermore, the Cartagena Protocol on Biosafety, to which the EU is a party, establishes international guidelines for the transboundary movement of GMOs [170].

The main limitations to date are regarding gaining approval from regulatory bodies, public perception, and potential safety risks that are difficult to quantify and predict, hereby requiring restrictive regulations. Transgenic plants might become invasive due to their competitive advantage against native species, disrupting the local biodiversity and potentially leading to economic and ecologic challenges [171]. The interaction of GEMs with other organisms may lead to unknown mutations by natural gene-transfer processes, with potential adverse impact on human health and the environment. Furthermore, GEMs may become dominant and affect the native microbial populations, potentially altering ecological functions [172]. In that sense, research is focusing on providing viable solutions. Xue et al. [173] developed an optimized, self-controlled circuit for effective mercury removal using an engineered *Pseudomonas putida* KT2440 strain. The model addresses complex aspects such as cell recovery, resistance, and biocontainment by implementing a suicide module following bioremediation, thus reducing the potential effect of the developed strain on the indigenous microbial community and ecological structure.

Genetic engineering presents a valuable opportunity to enhance bioremediation efforts against heavy metal contamination. By improving the natural capabilities of microorganisms and plants or introducing de novo traits, it can significantly boost xenobiotic removal. However, deploying genetically modified organisms beyond the lab requires a thorough understanding of the ecological impact and effective risk management.

### 4.2. Nano-Assisted Bioremediation: Synergistic Approaches for Environmental Restoration

**Figure 7:**
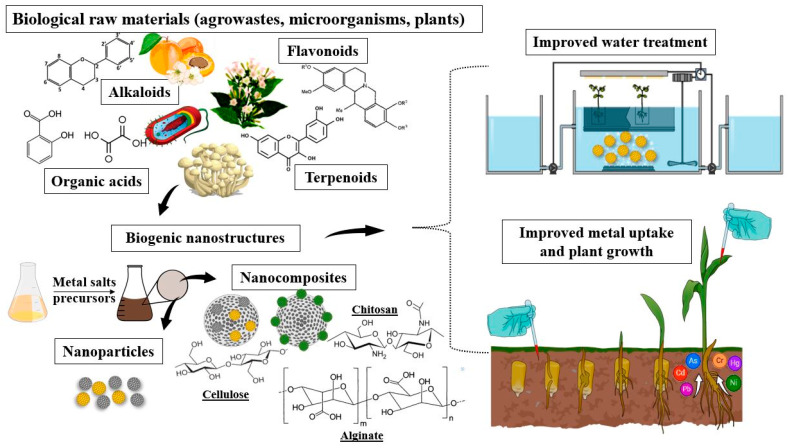
Biologically derived nanotechnologies for improving bioremediation efficiency in contaminated environments (figure created in the Mind the Graph platform).

Nanotechnology has become a rapidly evolving, cost-effective, and sustainable technology with potential benefits in agronomy, healthcare, industry, and environmental applications. The integration of nanomaterials into bioremediation strategies holds the potential for improvement through their distinctive characteristics of high surface area-to-volume ratio, reactivity, and catalytic capacity [173]. Additionally, nanomaterials may be modified and integrated into various polymers and matrices with tailored physico-chemical and mechanical properties, facilitating the development of novel, innovative solutions [174] (Figure 7). Engineered nanostructures such as nanoparticles, nanocomposites, nanofibers, and nanocatalysts can vary in shape, composition, reactivity, functionalization potential, and characteristics, thus possessing high selectivity for their target molecules [175].

Based on their primary components, nanomaterials may be categorized as metallic, bimetallic, or metal oxide nanoparticles, carbon-based materials, silica or zeolite-based materials, ceramic, polymeric or metal-organic frameworks, and bio-nanomaterials [176].

The biosynthesis of nanomaterials can waive the disadvantages of conventional production, therefore making the process more sustainable and cost-effective. Plant extracts and bacterial and fungal strains can be employed as reducing agents for metal salts, generating metal nanoparticles with higher stability [177]. The benefits consist of the absence of potentially toxic reagents without requiring additional stabilizers or capping agents, broad applicability, and increased bioavailability and biocompatibility [178]. Compared with metal nanoparticles obtained by conventional methods, biogenic NPs exhibit high colloidal stability as well as size and shape uniformity, their process of synthesis having the advantages of being controllable and easy to functionalize through genetic engineering [179]. Biosynthesis is carried out either intracellularly or extracellularly under the action of the enzymatic system which facilitates the precipitation and conversion of heavy metal ions into nanoparticles [180].

The formation of metal nanoparticles in the presence of heavy metals by microorganisms may also be a detoxifying mechanism. In the case of *Scwammiomyces occidentalis*, Cr(VI) was biotransformed to Cr(III) in the form of Cr_2_O_3_ NPs [181]. The same mechanism was observed in *Microbacterium* sp. MRS-1, capable of nickel bioremediation from contaminated industrial wastewater through the biosynthesis of unique floral-shaped NiO nanoparticles [182]. On the other hand, fungi produce bioactive compounds in substantial amounts and can withstand various conditions in bioreactor settings, thus making them more suitable for the large-scale production of metal NPs compared with bacteria. Additionally, even small amounts of fungal biomass can generate a high yield of nanoparticles [183]. Zhou et al. [184] concluded that enzymes such as NADPH-dependent oxidoreductases and cofactors were directly involved in the reduction of metal ions, leading to the formation of CuO nanoparticles by *Penicillium pimiteouiense*. Polyphenols, organic acids, phenolic acids, and proteins may also be involved in the stabilization and capping of metallic nanoparticles.

Several metal NPs such as gold, silver, cerium, palladium, zinc, selenium, iron, or platinum have been successfully synthesized using microbial or plant biomass and further used for the bioremoval of numerous pollutants, including heavy metals [185]. Among those, silver (AgNPs) and gold (AuNPs) nanoparticles have been extensively studied throughout the years compared to other metallic NPs, particularly for their excellent properties in electrochemistry, biomedicine, and biotechnology [186]. Foliar treatment of AgNPs synthesized using the extract of *Prunus dulcis* leaves significantly enhanced the phytoextraction of cadmium, arsenic, mercury, chromium, and copper in gold-mined soil, while simultaneously alleviating oxidative stress in *Abelmoschus esculentus* [187]. Nanoparticles of biological origin have been proven to alleviate stress in plants by modulating metabolic pathways. Biogenic selenium nanoparticles were found to inhibit the production of ROS in *Brassica napus* L. and improve basal metabolism under conditions of cadmium toxicity [188]. Additionally, metallic nanoparticles may improve plant growth, photosynthesis, and antioxidant enzymatic activity and even reduce heavy metal translocation, thus protecting aerial organs [189]. On the other hand, nanoparticles with proven antimicrobial efficiency such as AgNPs can disrupt the structure of the soil microbial community, shifting microbial diversity in favor of more resistant taxa, which could ultimately affect soil functions [190].

For the plant-mediated biosynthesis of nanoparticles, the use of low-cost precursors and common plant species makes the method desirable for large-scale production. Subterranean and aerial plant organs can be used, such as roots, stems, leaves, bark, flowers, fruits, or seeds [191], most of which could be agricultural wastes. Integrating them into the process of nanoparticle phytosynthesis for the generation of added-value products not only aids in the eco-restoration of polluted environments but also facilitates closing the loop of industrial activities for a circular economy approach. Different biodegradable wastes such as fruit and vegetable peels, eggshells, husks, and kernels have been used in the synthesis of metallic nanoparticles with efficiency in treating heavy metal contaminated wastewaters [192]. Biogenic iron nanoparticles produced using *Rosa indica* petals were used for the removal of Cr(VI) from wastewater [193]. Active phytochemicals such as flavonoids, phenolic acid, alkaloids, glycosides, and terpenoids were identified to be involved in the bioreduction of metals responsible for nanoparticle formation, as well as their stabilization and increased antimicrobial potential compared with bare metallic NPs [194].

Besides using nanoparticles generated by living organisms, biopolymers and nanocomposites can be assembled in order to be incorporated into bioremediation protocols. Fungal-derived chitosan can be integrated into nano-bio-composites with spectacular results in bioremediation [195]. A simple and cost-effective nanocomposite comprised of chitosan, graphene oxide, and zinc oxide biosynthesized by *Bacillus subtilis* embedded in a porous cryogel was successfully used for the bioremoval of cobalt from wastewater [196]. NiO nanoparticles obtained from *Salvadora persica* leaf extract have been used for the immobilization of laccase, achieving improved removal of phenols and antibiotics, increasing the stability and reusability of the enzyme [197]. Other sustainable materials, such as clay, sand, sediments, and natural zeolites, have also been used as nanocomposites with immobilized biogenic nanoparticles, allowing for the recovery and reuse of these materials. Their natural adsorbent capacity and accessibility makes them a viable option for the bioremediation of heavy metals in contaminated environments [198].

Although nano-assisted remediation is considered a cost-effective and easily scalable method [199], there are few studies that have analyzed the long-term economic implications compared with well-established chemical and physical synthesis methods to provide an accurate perspective. Correa et al. [200] performed a technical–economic analysis of bacterial-origin magnetite nanoparticles, revealing that base-case production costs can be higher than those required for chemical processes due to indirect operating costs such as maintenance, local taxes, and equipment depreciation, but genetically engineered biosynthesis might increase competitivity in the market.

In order to improve the cost-effectiveness of biogenic nanoparticle synthesis and determine the best upscaling option, El-Moslamy [201] obtained MgO-NPs using bacterial strains isolated from *Ocimum sancum*, an endemic plant of the study zone. Optimized biogenic nanoparticle synthesis and biomass production were achieved using a cost-effective multi-factorial approach and various scale-up strategies were tested, concluding that stirred-bioreactor fed-batch fermentation systems are the most efficient [201]. A more in-depth and substantial body of evidence is still needed to support the economical mass-production of biogenic nanoparticles in order to be able to replace conventional methods.

While nano-bioremediation shows great promise, the potential toxicity of nanomaterials to non-target organisms and their long-term accumulation must still be considered. As this field continues to evolve, establishing appropriate regulatory frameworks and management protocols is essential. Additionally, scaling up for commercialization requires long-term stability, reproducibility across laboratories, and reusability, challenges that biogenic nanomaterials still face.

## 5. Complexities and Constraints in Bioremediation: Addressing Key Scientific and Practical Challenges

Although bioremediation is becoming an established technology, there are still gaps in knowledge when it comes to the interactions between biological systems and pollutants on a larger scale, as well as the lack of a multidisciplinary approach for the effective restoration of contaminated environments in the long term [201]. The complexity of the pollution, as well as the inherently slow rate of the bioremediation process, requires a broader perspective on the particularities of individual contaminated sites and their interactions at the ecosphere level in order to choose the most appropriate method.

It is absolutely necessary to take into account several challenges, economic, social, and environmental, when it comes to extending to large-scale applications, one of the pressing issues, for example, being the appropriate disposal of biomass resulting from the bioremediation process. Furthermore, as decision-making is currently focused on mitigating pollution and finding climate change management measures, it is imperative to identify ongoing limitations and challenges that hinder sustainable development.

### 5.1. From Lab Controlled Experiments to Biosphere: Scaling up Bioremediation for Environmental Restoration to Real-World Implementation

The implementation of laboratory results on a large scale in the environment is generally hindered by complexity, the feasibility of the method, and the potential costs of failure [202], thus justifying the abundance of laboratory observations and microcosm configurations compared to the lack of long-term observations in the field. Large-scale biological remediation is most usually reserved for environmental disasters, in association with conventional methods [203]. Periodic research with applicability in natural environments may even be too costly. A study conducted by Ibáñez et al. [204] places the cost of in situ bioremediation for groundwater contaminated with arsenic through biostimulation and bioaugmentation for the duration of 2 years at 129,512.61 €, which may not be socio-economically feasible and requires further optimization in terms of production, operation, and construction for potential implementation.

The feasibility of field studies regarding the bioremediation of heavy metal-contaminated sites is also significantly influenced by environmental conditions. Process parameters can be easily controlled in the laboratory; hence, good performance might not be directly translated into real ecosystems.

In that sense, mesocosms represent a valuable option for bridging the gap between laboratory-scale observations and the complexity of the ecosystemic level. Odum defines mesocosms as “bounded and partially enclosed experimental setups, which simulate the natural environment, allowing for the simultaneous investigation of populations and ecosystems” [205]. As such, mesocosms are model ecosystems with controlled conditions, set in real environmental scenarios, which could therefore be useful in understanding the extended effects of xenobiotics as well as monitoring the multi-faceted aspects of bioremediation. Mesocosm settings were efficiently used to compare and assess the optimal bioremediation strategy for soils contaminated with high-molecular-weight hydrocarbons, revealing bioaugmentation as the most viable option, compared with biostimulation and natural attenuation [206]. Metagenomics were employed in a mesocosm setting for the development of an appropriate strategy in the case of marine sediments co-contaminated with polycyclic aromatic hydrocarbons, chromium, mercury, and zinc. The efficiency of three strategies was compared, namely single strain, mixed culture, and enriched natural consortia, concluding that the mixed culture approach has the highest potential to enhance the ability of the native microbial community to degrade target pollutants [207]. Furthermore, the influence of environmental conditions such as air temperature and soil characteristics such as the native microbial community structure, soil matrix structure, and physico-chemical particularities can be easily monitored in a mesocosm setup, thus determining the optimal adjustments to be made throughout the process [208].

Bioremediation is often considered a more cost-effective alternative to conventional physicochemical methods (such as chemical precipitation, ion exchange, membrane filtration, and adsorption) [209]. However, it must be kept in mind that the total cost depends on several factors, including the level of contamination, site conditions, treatment duration, and scalability.

An analysis of the costs involved in remediation processes using bioremediation and conventional methods can be carried out, taking into account several aspects:

- Initial investment and operational costs—Bioremediation involves lower setup and operational costs, especially for in situ applications (e.g., natural attenuation and bioaugmentation). It utilizes natural or engineered biological systems, reducing the dependency on expensive chemicals and high-energy processes. Depending on the type of bioremediation, costs may vary. For example, phytoremediation, although slower, may require up to 50% less cost compared with conventional treatments, while bioaugmentation requires more investments in process control, strain maintenance, and monitoring [210,211]. Physicochemical methods require high initial costs for equipment, chemicals, and infrastructure, as well as ongoing operational costs due to energy-intensive processes (e.g., electrochemical treatment or advanced oxidation). These methods generate secondary waste, requiring additional treatment and disposal procedures, thus increasing costs [212].
- Long-term sustainability and maintenance costs—Bioremediation tends to be more durable, with minimal long-term maintenance, but some methods such as phytoremediation require months to years to reach the desired level of removal, which can lead to increased monitoring costs. Physico-chemical methods provide rapid removal of heavy metals, but often require repeated applications for sustained results. These methods can degrade the structure of the ecosystem, requiring additional remediation costs [213].
- Cost of waste management—Bioremediation generates less hazardous waste compared to conventional methods, and in some cases the by-products obtained (e.g., biomass or biochar) can be reused for economic value (e.g., fertilizers and biofuels). Physico-chemical methods produce large volumes of toxic sludge or secondary pollutants, which require significant costs for handling, transportation, and disposal [214].
- Cost-effectiveness on a large scale—Bioremediation has the potential to be more economically feasible for large-scale contamination scenarios in integrated practices, both for long-term treatments as well as environmental restoration. Physico-chemical methods, although suitable for highly contaminated sites where rapid treatment is needed, require high costs that make them disadvantageous for long-term remediation [215].

A cost-effectiveness analysis conducted by Chen and Li [216] revealed that biological remediation technologies such as phytoremediation might be more costly than conventional physical treatments, as more rounds may be needed for more heavily contaminated sites, requiring long-term management.

Bioremediation is generally considered more cost-effective than physicochemical methods, especially for large-scale, long-term applications, due to lower operational, waste management, and sustainability costs. However, as physicochemical methods provide faster results, they may be preferred in cases of acute contamination requiring immediate intervention.

A hybrid approach which combines chemical pretreatments or physical techniques followed by suitable biological remediation technologies could be more favorable in terms of costs and efficiency for complete detoxification. On the other hand, Wan et al. [217] estimate a lower cost of phytoremediation compared with conventional methods, with the long-term benefits such as ecosystem service function, decrease in income loss, and improved agricultural production being more valuable. In addition, the accurate prediction or prevention of unexpected events that may hinder the activity of the biological agents employed, as well as improving the level of mechanization, will contribute to lowering the overall costs of bioremediation.

### 5.2. Management of Bioremediation Waste

As the importance and efficiency of biological remediation gains more recognition, a significant issue arises in regards to the sustainable disposal and treatment of biomass derived from these methods. Conventional disposal and volume reduction through landfill dumping or incineration are associated with the emission of hazardous particles and gases that might contain volatile compounds, thus posing health risks for the human population as well as potentially reintroducing pollutants into the environment [218]. Thermal conversion, through pyrolysis, or biochemical conversion, through anaerobic digestion or composting, may not only be efficient options for waste disposal in a sustainable, energy-efficient, and ecological manner but also for the potential of obtaining value-added products [219].

Pyrolysis is regarded as a sustainable method for the conversion of organic matter into biofuels, biochar, and biogas. Based on the heating rate, the process is categorized as slow, fast, or flash, the use of lower temperatures favoring char production while higher temperatures maximize biofuel production [220]. Biochar has the highest potential to further support environmental aspects, as heavy metals can be stabilized within its structure through slow pyrolysis, which is carried out at low temperatures and low heating rates. The resulting product could then be valorized for contaminant removal in aquatic environments, carbon sequestration strategies, and generation of energy, as well as a fertilizing agent [221]. Biochar can modify soil pH and cation exchange capacity while also increasing total organic carbon content, reducing the mobility of metal ions, and supporting soil functions [222]. Flash and slow pyrolysis were compared as treatment options for *Sorghum bicolor* L. biomass after the phytoextraction of Ni and Zn. Slow pyrolysis was more favorable for heavy metal recovery in the resulting char, thus producing bio-oil without the extracted pollutants. Furthermore, the resulting biochar may be used for energy purposes with the addition of gas filters to trap the heavy metals [223]. Pyrolysis was observed to stabilize heavy metals present in the residual plant biomass, with a decrease in the bioavailable, exchangeable fraction.

It has also been observed that the concentration of toxic metals with leaching potential is under the required limit, making pyrolysis products, especially biochar, options with low potential ecological risks [224]. The main concerns regarding pyrolysis have been greenhouse gas (GHG) emissions. By optimizing the process parameters, particulate matter emissions, along with carbon monoxide, nitrogen oxide, and sulfur dioxide emissions, can be significantly reduced compared to emissions generated by diesel fuel incineration, thus falling within the standard requirements [225]. The direct application of biochar obtained from residual phytoremediation biomass onto the soil may improve crop yield, soil fertility, and microbial activity [221], therefore improving soil function as a reservoir in the biogeochemical cycle of nutrients. Moreover, a combined method of composting with the addition of pyrolysis products could increase the quality of both components, thus enhancing their effects, as well as generate economic benefits and improvements in living standards [226].

Anaerobic digestion and composting are also promising options for the management of bioremediation wastes, especially for smaller-scale disposal. Anaerobic digestion is efficient for biogas production through the decomposition of organic matter by anaerobes [227] as well as solubilization and recovery of heavy metals from plant biomass residues obtained after phytoremediation [228]. Composting can be combined with anaerobic digestion for energy production and material recovery, but its most recognized utility is for the production of biofertilizers, with significant importance in agriculture [229].

The main concern remains regarding the safety of utilizing these products in terms of heavy metal remobilization. A pilot study performed by Song [230] tested the feasibility of using plant biomass produced after phytoremediation for composting purposes, consisting of mixing the biomass with sewage sludge containing heavy metals. It was observed that heavy metal concentration decreased in the sludge, applying compost onto soil did not lead to an increase in the concentration of the target metals, and bioaccumulation was not significant in the plant species later used for soil reclamation. Anaerobic digestion was also proven to be efficient in treating heavy metal-containing plant biomass, with significant production of biogas [231]. By using energy crops, a dual issue is addressed, concerning the management of agricultural species used in phytoremediation or simply grown in soils contaminated with heavy metals.

Biomass disposal technologies offer numerous advantages in terms of efficiency, resource use, and environmental impact, addressing an urgent problem of taking on the responsibility of secondary waste resulting from bioremediation. However, further research is still needed for the optimization and proper implementation of these technologies, to prevent leaching the heavy metals from the residual biomass into the environment again. These methods may not be suitable for biomass heavily contaminated with heavy metals as particularities such as soluble metal concentration and metal speciation may exert an inhibitory or even bactericidal effect on the active bacterial communities, thus affecting process performance [232].

### 5.3. Public Perception and Acceptance from Scientific Innovation to Societal Expectations—Challenges and Pathways Forward

The successful implementation of bioremediation strategies depends not only on scientific and technological advances, but also on societal perception and acceptance. Despite its potential as an environmentally friendly and cost-effective approach to environmental restoration, bioremediation often faces skepticism due to concerns about its efficacy, safety, and long-term impact. Public concern may stem from lack of awareness, misinformation, or perceived risks associated with genetically modified organisms (GMOs) and use of microbial strains. Furthermore, regulatory constraints and the absence of clear communication between scientists, policymakers, and local communities often hinder the widespread adoption of this method. To bridge this gap, transparent risk assessment, public engagement initiatives, and educational programs are essential. Additionally, building trust through community engagement, demonstrating successful case studies, and aligning bioremediation efforts with sustainability goals can foster greater societal acceptance and pave the way for its large-scale application.

The social factor is one of the most important drivers influencing the large-scale implementation of bioremediation practices and ultimately the fate of the environment. Public acceptance regarding more sustainable management practices for pollution needs to be acknowledged and taken into consideration, as it is vital for implementing, improving, and adjusting technologies related to contaminated areas. A lack of understanding within the community may hinder the ability to successfully address the environmental impact of hazardous compounds and may also influence decisionmakers and, consequently, regulations. A survey was conducted by Prior [233] on residents living in the proximity of thirteen contaminated sites across Australia, revealing that acceptance is significantly influenced by subjective perception of the risks of pollution and benefits of targeted remediation technologies. Socio-demographic factors such as age, education level, and previous experience with environmental issues also correlate with varying levels of acceptance. Hence, a lack of understanding and failure of concise, efficient communication can hinder the implementation of remediation technologies in real contaminated environments as well as the integration of novel technologies such as synthetic biology or nanotechnologies.

Generally, the public is receptive towards technologies which pose less of an impact onto the environment. Public understanding towards environmental biotechnology has improved in the last two decades, yet emerging technologies such as gene editing are still under scrutiny. Jha et al. [234] propose a number of measures to improve public perception in matters of environmental targeted technologies, such as ensuring better interactions between researchers and industries, dissemination of information tailored for all social levels, and structuring a business environment for small and medium-sized business to encourage shifting towards greener products and services, as well as coordination between the main bodies of authority responsible for law enforcement and research in order to strengthen public trust and awareness.

### 5.4. Future Trends in Bioremediation

Taking into consideration the aspects presented in this paper, future studies should focus on expanding biological remediation approaches while assessing their potential ecological impact, integrating biotechnological innovations, and opting for interdisciplinary approaches in order to increase efficiency and applicability in the field. Thus, we can summarize some trends:

- Genetically modified microorganisms (GEMs)—Through advances in biology and genetic engineering, microorganisms with greatly improved metal uptake, resistance, and degradation capabilities have been developed. Novel technologies such as CRISPR and gene editing offer the possibility of optimizing microbial metabolism to create highly specific and efficient mechanisms for bioremediation.
- Nanotechnology—The integration of nanomaterials, such as bio-nanocomposites and biogenic nanoparticles that can stabilize and immobilize metals, is another approach to stimulate microbial activity and control the bioavailability and mobility of metals for efficient remediation;
- Microbial consortia with complementary metabolic pathways can contribute to improved sequestration and degradation of heavy metals;
- Enhancing phytoremediation and rhizoremediation by using genetically modified plants and exploiting plant-microbe interactions represents a sustainable solution, especially for large-scale and in situ applications;
- Electro-bioremediation via integrating electrochemical systems with microbial cells is another approach to improve metal recovery while generating bioenergy, thus leading to a more cost-effective and sustainable remediation process;
- AI (Artificial Intelligence) and Big Data in bioremediation enable AI-based modeling and machine learning to optimize selection programs for microbial strains or plants, predict remediation efficiency, and design large-scale bioremediation strategies with high accuracy and optimized conditions.

The overall goal of all these strategies is field applications on a large scale. As can be seen, research is moving towards real-world applications, focusing on pilot and full-scale implementation of bioremediation technologies in contaminated sites, respecting and ensuring regulatory compliance and long-term ecological safety. All these trends, along with the undeniable advancement of nanotechnology and bioengineering, only confirm that bioremediation will represent a solution of the future, moving towards more efficient, more cost-effective, and more environmentally sustainable solutions for mitigating heavy metal contamination.

## 6. Conclusions

This review highlights the remarkable advancements in bioremediation as a viable, eco-friendly, and efficient strategy for mitigating heavy metal pollution, supporting environmental restoration, and promoting sustainable development. Biological remediation harnesses living organisms to degrade, stabilize, and transform pollutants, including heavy metals and metalloids such as arsenic, lead, cadmium, arsenic, and mercury, with minimal ecological disruption. However, despite its advantages, there are several challenges hindering its large-scale application. These include the need for multiple treatment cycles in highly contaminated sites, the impact of site-specific physicochemical conditions on microbial and plant metabolic efficacy, the financial burden of long-term treatments at larger scales, and the safe disposal of residual biological waste generated during treatment.

Ongoing research continues to refine bioremediation strategies, improving their efficiency and scalability, while deepening our understanding of the metabolic pathways and ecological interactions governing these processes. Emerging technologies, such as nano-enhanced bioremediation and genetically engineered organisms, represent transformative approaches, offering enhanced control over metabolic activities and introducing innovative materials for more efficient heavy metal sequestration. However, challenges related to societal perception, regulatory constraints, and potential environmental risks must be carefully addressed to ensure the responsible and ethical deployment of these novel remediation strategies.

A future-oriented perspective underscores the importance of a multidimensional approach that integrates bioremediation methods tailored to the complexity of each contaminated site. Numerous studies have already proven the efficiency of bacteria, fungi, microalgae, and plants, either individually or in consortia, to remove heavy metals from a variety of media. To scale up these solutions, long-term field applications must be reinforced through interdisciplinary collaborations between policymakers, scientists, industry stakeholders, and the public, ensuring that scientific advancements translate into effective policies and infrastructure investments.

Moreover, the integration of organic waste from economic sectors and bioremediation by-products into value-added applications, such as biochar production, biofertilizers, and metal recovery technologies, aligns with circular economy principles, further enhancing the sustainability of remediation strategies.

In conclusion, bioremediation is evolving into a well-established, adaptable, and highly promising solution for the sustainable management of heavy metal pollution. By leveraging targeted remediation methods based on specific heavy metal contaminants and fostering a collaborative framework, its continued advancement will contribute to a more balanced coexistence between humanity and the natural environment.

## Figures and Tables

**Table 1:** Maximum contamination levels (MCLs) established by the WHO in water and agricultural soil, respectively, by the United States Environmental Protection Agency (US-EPA) in water and sludge for agricultural use.

Elements	Water		Soil/Sludge Applied to Soil	
	WHO Limits (mg/L)	US-EPA Limits (mg/L)	WHO Limits (mg/kg d.w)	US-EPA Limits (mg/kg d.w)
Arsenic (As)	0.01	0.01	20	0.39
Cadmium (Cd)	0.003	0.005	3	1.4
Total Chromium (Cr)	0.05	0.1	100	100
Cobalt (Co)	0.005	-	50	7.5
Lead (Pb)	0.01	0.015	50	400
Inorganic Mercury (Hg)	0.006	0.002	0.004	0.2
Nickel (Ni)	0.07	0.1	100	75

d.w means dry weight.

**Table 2:** Current remediation methods used and reported for the treatment of terrestrial and aquatic environments contaminated with heavy metals.

Remediation Methods	Technologies	Environment	Advantages	Limitations	Ref.
Physical	− Flotation − Aeration − Thermal treatment − Adsorption − Membrane/filter separation	Aquatic	− Simple and flexible technology; − Adaptable to several treatment formats; − Low generation of solid wastes compared to other methods; − Controllable processes.	− Requires high costs and energy consumption; − Need for chemical treatment for regeneration and improvement; − Performance depends on the type of material.	[64]
	− Surface capping − Encapsulation − Landfilling − Thermal desorption − Vitrification − Electrokinetic extraction	Terrestrial	− Simple application; − Low costs; − Can eliminate the risk of exposure.	− Limited to small areas; − Low efficiency for highly contaminated areas; − Loss of soil environmental functions; − Dependent on soil particularities; − Can be time consuming (up to several years); − Does not always remove pollutants, can merely isolate the contaminated area.	[65]
Chemical	− Precipitation − Coagulation/Flotation − Ion exchange − Electrochemical technologies − Reverse osmosis − Photocatalysis	Aquatic	− Easily automated; − Simple operation; − Relatively economic; − High removal efficiency.	− Can be highly sensitive to pH; − Depending on method, it can require high costs and can be incomplete in removal; − Production of sludge that needs additional treatment.	[66]
	− Immobilization − Stabilization − Chemical leaching − Permeable reactive barriers − Ion exchange resins − Oxidation/Reduction	Terrestrial	− Minimal soil disturbance; − High removal efficiency; − Adaptable for high pollutant loads.	− Can require high costs; − Dependent on soil particularities; − Performance influenced by the type of reagents; − Does not completely eliminate contaminants from the area; − Can be time consuming (up to several years); − Generates secondary pollution in the form of chemical wastes.	[67]
Biological	**Phytoremediation** − Rhizofiltration − Phytoextraction − Phytostabilization − Phytovolatilization **Bioremediation** − Biosorption − Activated sludge − Aerobic granular systems − Bioreactors	Aquatic	− The residual biomass can be repurposed for value-added products such as biogas and biochar; − Supports ecosystem functions; − Low environmental impact.	− The residual biomass needs additional treatment; − May require combined methods to be efficient; − May require a long time to be effective.	[68]
	**Bioremediation**: − Bioaugmentation − Bioattenuation − Biostimulation − Bioslurping − Bioventing − Biosparging − Land farming **Phytoremediation**: − Phytoextraction − Phytodegradation − Rhizofiltration − Phytovolatilization	Terrestrial	− Suitable for a wide range of contaminants; − Simple to implement and monitor; − Easy to control in bioreactors; − Can remove pollutants from the environment and reduce their toxicity; − Conservation of soil structure and functions; − The residual biomass can be repurposed for biogas and biochar production.	− Requires the acclimatization of biological systems used; − May require long time to be effective; − May require combined methods.	[69]

**Table 3:** Microbial strains proven to accumulate heavy metals and their removal efficiency (%).

Microbial Strains	Heavy Metals	Removal Efficiency (%)	Initial Concentration (mg/L)	Experiment Duration	Treated Media	Reference
**Bacteria**						
*Alteromonas macleodii*	Pb Ni Cd	73.8 54 53	200	10 h	Synthetic broth	[86]
*Cloacibacterium normanense*	Ni	85	48	2.5 h	Municipal wastewater	[87]
*Ochrobactrum intermedium*	Pb Ni	85.34 74.87	100	3 days	Aqueous solution	[88]
*Ochrobactrum ciceri*	Pb Ni	71.20 88.48	100	3 days	Aqueous solution	[88]
*Cutibacterium* sp.	Pb	35.19	170	7 days	Aqueous solution	[89]
*Lactobacillus plantarum*	Cd Pb Ni Cr	100 100 100 100	-	1 h	Battery-manufacturing effluent	[90]
*Bacillus megaterium*	Pb Ni Cd	10.54 73.02 24.68	3200 3200 3200	4 days	Aqueous solution	[91]
*Rhizopus stolonifera*	Pb Ni Cd	23.79 58.89 17.06	3200 3200 800	4 days	Aqueous solution	[91]
*Cupriavidus necator*	Pb Cr	63.56 81.32	0.247 1.82	5 days	Soil	[92]
*Pseudomonas putida*	Pb Cr	83.81 80.77	0.247 1.82	5 days	Soil	[92]
**Fungi**						
*Aspergillus niger*	Cr Co As Pb Cd	100 71.4 69 59 57	50 100 1 100 5	7 days	Wastewater	[93]
*Aspergillus fumigatus*	Pb Cr Ni	99 75 100	30	3 days	Aqueous solution	[94]
*Beauveria bassiana*	Cd Cr Ni	63.4 61.13 75	30	5 days	Aqueous solution	[95]
*Trichoderma harzianum*	Cd Pb Ni	98.63 84.50 69.07	2.19 2.69 -	28 days	Wastewater	[96]
*Saccharomyces cerevisiae*	Cd	69.56	500 500	5 days	Contaminated soil	[97]
*Bacillus subtilis*	Cd	75.76	500 50	5 days	Contaminated soil	[97]
*Candida lipolytica*	Pb	30	1000	120 days	Synthetic wastewater	[98]
*Rhodotorula mucilaginosa*	Pb	25.2 40.6 32.6 25.24	500 1000 2000 2500	3 days	Aqueous solution	[99]
**Consortia of microorganisms**						
*Aspergillus fumigatus* and *Aspergillus terreus*	Cd Cr Pb	93.28 89.41 97.13	-	6 days	Tannery effluent	[100]
*Perenniporia* *subtephropora*, *Daldinia starbaeckii*, *Phanerochaete* *concrescens*, *Cerrena aurantiopora*, *Fusarium equiseti*, *Polyporales* sp., *Aspergillus niger*, *Aspergillus fumigatus*, *Trametes versicolor*	As Cr	62 42	-	100 days	Soil	[101]
*Paecilomyces lilacinus*, *Antrodia serialis*, *Penicillium cataractum*	As Cr	48 36	-	100 days	Soil	[101]
*Sphingomonas* *paucimobilis*, *Rhizobium radiobacter*, *Bacillus subtilis*, *Bacillus pumilus*	Pb Cd Cr	98.08 95.43 97.12	0.314 0.285 0.174	4 days	Industrial wastewater	[102]

**Table 4:** Different plant species acting as hyperaccumulator for heavy metals and their removal efficiency (%).

Plant Species	Heavy Metals	Removal Efficiency (%)	Initial Concentration (mg/L)	Experiment Duration	Treated Media	Reference
*Sorghum bicolor*	Ni	97.28	25	20 min	Aqueous solution	[128]
	Cr	99.8	5	30 min		
*Helianthus annuus*	Pb	70.88	10	4 weeks	Contaminated soil	[129]
*Hydrangea paniculata*	Pb	50.65	10	4 weeks	Contaminated soil	[129]
*Echinochloa pyramidalis*	Cd Ni Pb	37.99 22.25 88.74	5	6 weeks	Soil contaminated with wastewater	[130]
*Ludwigia stolonifera*	Cd Ni Pb	48.04 32.3 84.29	5	6 weeks	Soil contaminated with wastewater	[130]
*Tagetes patula*	Cd Cr Pb	31.61 47.56 94.99	0.715 0.513 1.098	8 weeks	Contaminated river water	[131]
*Portulaca grandiflora*	Cd Cr Pb	55.94 18.52 92.81	0.715 0.513 1.098	8 weeks	Contaminated river water	[131]
*Bassica scoparia*	Cd Cr Pb	100 26.12 93.72	0.715 0.513 1.098	8 weeks	Contaminated river water	[131]
*Ricinus communis*	Cr Cd Ni Pb	34.48 99.89 48.27 53.43	0.002 0.019 0.014 0.018	Not specified	Distillery sludge	[132]
*Eichhornia crassipes*	Cr	99.98	10.4749	3 h	Tannery effluent	[133]
*Lemmna minor*	Cd Cr Ni Pb	44.93 32.26 74.48 79.1	0.0227 0.5252 0.1117 0.2526	5 weeks	Industrial wastewater from tannery and battery industries	[134]
*Phragmites australis*	Cd Cr Ni Pb	43.3 51.2 55.8 45.7	0.079 0.142 0.088 0.060	2 weeks	Urban sewage mixed with industrial effluent	[135]
*Typha latifolia*	Cd Cr Ni Pb	39.7 45.6 51.1 40	0.079 0.142 0.088 0.060	2 weeks	Urban sewage mixed with industrial effluent	[135]
Consortia of plants						
*Phragmites australis* and *Typha latifolia*	Cd Cr Ni Pb	60 68.1 73.8 61	0.079 0.142 0.088 0.060	2 weeks	Urban sewage mixed with industrial effluent	[135]
*Echinochloa pyramidalis* and *Ludwigia stolonifera*	Cd Ni Pb	61.52 27.8 93.22	5	6 weeks	Soil contaminated with wastewater	[130]

## Data Availability

No new data were created or analyzed in this study.
